# CdGAP/ARHGAP31, a Cdc42/Rac1 GTPase regulator, is critical for vascular development and VEGF-mediated angiogenesis

**DOI:** 10.1038/srep27485

**Published:** 2016-06-07

**Authors:** Christine Caron, Jonathan DeGeer, Patrick Fournier, Philippe M. Duquette, Vilayphone Luangrath, Hidetaka Ishii, Fereshteh Karimzadeh, Nathalie Lamarche-Vane, Isabelle Royal

**Affiliations:** 1 CRCHUM - Centre de recherche du Centre Hospitalier de l’Université de Montréal and Institut du Cancer de Montréal, Montréal, Québec, Canada; 2 Cancer Research Program, Research Institute-McGill University Hospital Centre, Montreal, Quebec, Canada; 3 Department of Anatomy and Cell Biology, McGill University, Montreal, Quebec, Canada; 4 Department of Medicine, Université de Montréal, Montréal, Québec, Canada

## Abstract

Mutations in the *CdGAP/ARHGAP31 gene*, which encodes a GTPase-activating protein for Rac1 and Cdc42, have been reported causative in the Adams-Oliver developmental syndrome often associated with vascular defects. However, despite its abundant expression in endothelial cells, CdGAP function in the vasculature remains unknown. Here, we show that vascular development is impaired in CdGAP-deficient mouse embryos at E15.5. This is associated with superficial vessel defects and subcutaneous edema, resulting in 44% embryonic/perinatal lethality. VEGF-driven angiogenesis is defective in CdGAP^−/−^ mice, showing reduced capillary sprouting from aortic ring explants. Similarly, VEGF-dependent endothelial cell migration and capillary formation are inhibited upon CdGAP knockdown. Mechanistically, CdGAP associates with VEGF receptor-2 and controls VEGF-dependent signaling. Consequently, CdGAP depletion results in impaired VEGF-mediated Rac1 activation and reduced phosphorylation of critical intracellular mediators including Gab1, Akt, PLCγ and SHP2. These findings are the first to demonstrate the importance of CdGAP in embryonic vascular development and VEGF-induced signaling, and highlight CdGAP as a potential therapeutic target to treat pathological angiogenesis and vascular dysfunction.

Expansion of the vascular system is essential for organ growth during development as well as during tissue repair. However, abnormal vessel growth after birth supports the progression of various pathologies including cancer, ischemic diseases and chronic inflammation^1^,^2^,^3^,^4^,^5^. Since the molecular mechanisms underlying vascular development have not been fully described, the identification of novel targets may lead to the discovery of improved therapeutics for the treatment of these diseases. Vasculogenesis initiates around embryonic day (E) 8.5, with the assembly and differentiation of mesoderm-derived endothelial progenitor cells that form the primitive vascular plexus^6^,^7^,^8^. This process is followed by angiogenesis, which is the formation of new blood vessels budding and sprouting from the pre-existing primitive vessels^7^,^9^. Angiogenesis is driven by endothelial cell activation, migration and invasion, which together support the elongation of newly formed vascular tubes to generate complex vascular networks of arteries and veins^10^.

Of the growth factors involved in vessel formation, vascular endothelial growth factor (VEGF) acts as a key regulator of vasculogenesis and angiogenesis through its tyrosine kinase receptors Flt-1 (VEGFR1) and Flk-1 (VEGFR2)^7^. VEGFR2 is however the main receptor promoting VEGF-induced endothelial cell proliferation, migration, survival, and permeability through the concerted activation/phosphorylation of multiple signaling effectors, including the proximal scaffolding adapter protein Gab1, Akt, Src, PLCγ and ERK1/2^11^,^12^,^13^,^14^,^15^,^16^,^17^. In addition, the Rho family GTPase Rac1 plays a central role in the formation of blood vessels by promoting the migration, adhesion, sprouting, and permeability responses of endothelial cells to VEGF^18^,^19^,^20^,^21^. Notably, the endothelial cell-specific deletion of the *Rac1* gene upon Tie2 promoter-driven Cre expression leads to embryonic lethality around E9.5 and severe defects in vascular and cardiac development, with a complete lack of small branched vessels^19^. A role for Rac1 in postnatal angiogenesis was further shown in endothelial cell-specific haploinsufficient mice^21^. Thus, Rac1 is a critical promoter of vascular development, likely through the regulation of endothelial cytoskeletal remodelling.

Rac1, cycling between an active GTP-bound and inactive GDP-bound conformation is tightly controlled by three classes of regulators, namely guanine nucleotide exchange factors (GEFs), GTPase-activating proteins (GAPs), and GDP dissociation inhibitors (GDIs)^22^,^23^. However, despite the abundance and importance of these regulators in the spatio-temporal control of Rac1 function, little is known about their implication during vascular development^24^. In particular, CdGAP (Cdc42 GTPase-activating protein)/ARHGAP31, a GAP for Rac1 and Cdc42^25^,^26^, was reported to be highly expressed in human umbilical vein endothelial cells (HUVECs)^24^, and to be regulated in *Zebrafish* by the transcription factor Ets, a master regulator of angiogenesis^27^,^28^. Outside of the vascular system, pro-migratory and pro-invasive functions were ascribed to CdGAP, which was shown to regulate directional membrane protrusions of migrating osteosarcoma cells^29^,^30^,^31^ and TGFβ-dependent cell motility and invasion of breast cancer cells^32^. Recently, truncating mutations in the terminal exon of the *CdGAP* gene were identified in patients with a rare developmental disorder, Adams-Oliver Syndrome (AOS)^33^,^34^, which is frequently associated with cardiac and vascular anomalies^35^,^36^. We show here that CdGAP-deficient embryos exhibit incompletely penetrant embryonic lethality, edema, and vascular defects. Importantly, VEGF-mediated cell signaling, migration, and capillary formation are impaired in CdGAP-depleted endothelial cells. Collectively, these results demonstrate a critical role for CdGAP in vascular development and VEGF-dependent angiogenesis, and provide further insights into the molecular causes of AOS.

## Results

### CdGAP depletion causes incompletely penetrant embryonic lethality

**Figure 1:**
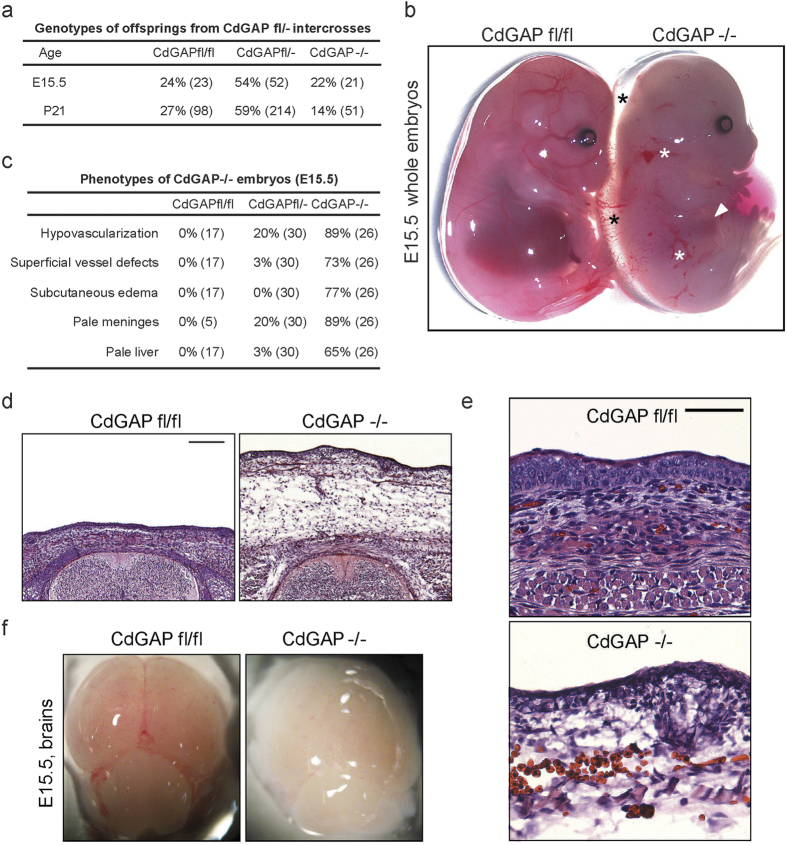
CdGAP^−/−^ mice exhibit incompletely penetrant embryonic lethality, edema, and vascular defects. (**a**) Breeding table from heterozygous intercrosses shows that CdGAP^−/−^ mice were not born at the expected Mendelian ratio, exhibiting 44% embryonic/perinatal lethality. Numbers in parentheses indicate the numbers of embryos (E15.5) or born mice (P21) (P = 6,4429E-06 (χ^2^ test)). (**b**) At E.15.5, CdGAP^−/−^ whole embryos displayed vascular defects with subcutaneous edema (black asterisks), various degrees of subcutaneous hemorrhages (white asterisks), and a pale liver (white arrowhead) compared to control CdGAP^fl/fl^ embryos. (**c**) Percentage of control CdGAP^fl/fl^, heterozygous CdGAP^fl/−^, and CdGAP^−/−^ embryos showing hypovascularization, vascular defects and edema at E15.5. Numbers in parentheses indicate the numbers of embryos. (**d,e**) H&E stainings of transverse sections of E15.5 CdGAP^fl/fl^ and CdGAP^−/−^ embryos. Note edema (**d**) and infiltrating red blood cells (**e**) in subcutaneous regions of CdGAP^−/−^ embryos compared to control. Representative images of at least 3 embryos per genotype. Scale bars, 200 μm (**d**) and 50 μm (**e**). (**f**) Brains dissected from E15.5 CdGAP^fl/fl^ and CdGAP^−/−^ embryos. Note the hypovascularized, pale meninges of the CdGAP^−/−^ mice.

To explore the role of CdGAP during embryonic development, we generated a CdGAP-deficient mouse model. First, we designed a conditional floxed exon 1 allele to remove the ATG start codon of the *CdGAP* gene (Supplementary Fig. 1a). The conditional CdGAP^fl/fl^ mice were then crossed with mice expressing Cre recombinase under the Meox2 promoter, which is active as early as E5 in epiblast-derived tissues^37^. Next, to eliminate the possibility of non-specific effects caused by Meox2-Cre recombinase expression, heterozygous CdGAP^fl/−^; Meox2-Cre^−^ mice were intercrossed to generate CdGAP^−/−^ mice, as assessed by PCR (Supplementary Fig. 1b). The complete absence of CdGAP expression in CdGAP^−/−^ mice was confirmed by western blotting of protein lysates of lung, brain, and heart tissues compared to those isolated from CdGAP^fl/fl^ mice (Supplementary Fig. 1c). CdGAP^−/−^ pups initially seemed morphologically indistinguishable from control CdGAP^fl/fl^ pups. However, they were not born at the expected Mendelian ratio, and showed 44% lethality by post-natal day (P) 21 (Fig. 1a). Thus, the complete loss of CdGAP expression leads to incompletely penetrant embryonic/perinatal lethality.

### CdGAP-deficient embryos display vascular defects and edema

To better evaluate the potential cause of lethality of CdGAP-deficient mice, we examined E15.5 CdGAP^−/−^ whole embryos, which were obtained at the expected Mendelian ratio (Fig. 1a). Intriguingly, hypovascularization was apparent in 89% of CdGAP-depleted embryos, which were paler in appearance, and in 20% of heterozygous CdGAP^fl/−^ embryos (Fig. 1b,c). This was accompanied by progressive superficial vessel defects of varying severity as defined by the presence of hemorrhages (white asterisks) in 73% of CdGAP-deficient embryos and 3% of CdGAP^fl/−^ embryos (Fig. 1b,c). This was further evidenced by the development of prominent subcutaneous edema (black asterisks) in 77% of CdGAP-deficient embryos (Fig. 1b–d) and the infiltration of red blood cells into the subcutaneous regions of CdGAP^−/−^ embryos (Fig. 1e). Furthermore, hypovascularization was also evident in the meninges surrounding the brains dissected from CdGAP^−/−^ embryos (Fig. 1c,f) and the noticeably paler livers (white arrowhead) of CdGAP-deficient embryos (Fig. 1b,c). Taken together, these results indicate that CdGAP plays a key role in vascular development during embryogenesis.

### CdGAP is required for VEGF-mediated angiogenesis

**Figure 2:**
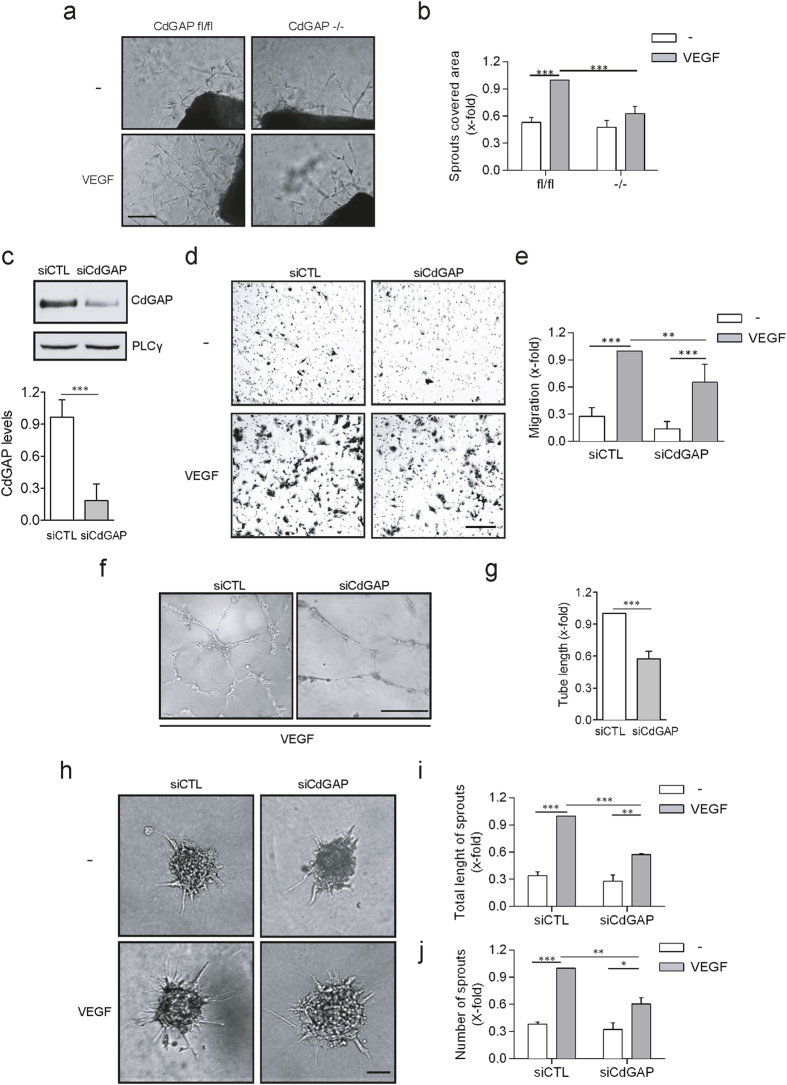
CdGAP is required for VEGF-mediated angiogenesis. **(a)** Aorta rings prepared from adult CdGAP^fl/fl^ and CdGAP^−/−^ mice were embedded in Matrigel and cultured in medium containing either VEGF (50 ng/ml) or PBS (−) as a control. Scale bar, 200 μm. (**b**) The average surface occupied by capillaries sprouting from the aortic rings was quantified using the Metamorph software. n = 5/genotype per condition. Error bars indicate SEM (***P ≤ 0.001, One-way ANOVA, Newman-Keuls post-test). (**c**) Western blot analysis of CdGAP expression levels in HUVECs electroporated with control siRNAs (siCTL) and CdGAP siRNAs (siCdGAP). PLCγ was used as a protein loading control. CdGAP expression levels were quantified by densitometric analysis. TCL, total cell lysate. n = 4, error bars indicate SEM (***P ≤ 0.001, Unpaired student’s t-test). (**d**) Control and CdGAP-depleted HUVECs plated on gelatin-coated filters were subjected to a Boyden Chamber migration assay with or without VEGF (10 ng/ml). Scale bar, 100 μm. (**e**) Ratio of the average number of migrating cells normalized to the number of VEGF-stimulated migrating control cells from 4 independent experiments. Error bars indicate SEM (**P ≤ 0.01, ***P ≤ 0.001, One-way ANOVA, Bonferroni’s multiple comparisons post-test). (**f**) Control and CdGAP-depleted HUVECs were trypsinized and plated on solid Matrigel in the presence of VEGF (50 ng/ml). Scale bar, 100 μm. (**g**) Ratio of total capillary tube length from 3 independent experiments. Error bars indicate SEM (***P ≤ 0.001, Unpaired student’s t-test). (**h**) Control and CdGAP-depleted HUVECs grown as spheroids were embedded in collagen containing either VEGF (50 ng/ml) or PBS (−) as a control. Scale bar, 100 μm. (**i,j**) Ratio of the total length (**i**) and number (**j**) of sprouts. Error bars indicate SEM (n = 4, **P ≤ 0.01, ***P ≤ 0.001, One-way ANOVA, Bonferroni’s multiple comparisons post-test).

Due to the pronounced vascular deficits observed in CdGAP-null embryos, we next examined whether CdGAP was involved in the promotion of VEGF-induced angiogenesis. To test this, aortas from surviving 6 week-old CdGAP^−/−^ or control mice were harvested and embedded in Matrigel to analyze VEGF-dependent angiogenic sprouting. In this context, VEGF induced a two-fold increase in the growth of capillary sprouts from the aortic rings of control mice compared to the unstimulated condition (Fig. 2a,b). In striking contrast, VEGF failed to significantly induce the growth of capillary sprouts from CdGAP-null aortic rings (Fig. 2a,b). These results demonstrate that CdGAP is essential for VEGF-mediated capillary protrusion and elongation in the adult mouse *ex vivo*.

### CdGAP is required for VEGF-induced endothelial cell migration and capillary formation

We next investigated if endothelial CdGAP was involved in the promotion of cell migration and capillary formation in response to VEGF *in vitro*. CdGAP expression was downregulated in HUVECs electroporated with siRNAs targeting the 5′UTR of human CdGAP^29^ (Fig. 2c), and Boyden chamber migration assays were first performed. VEGF induced a 3-fold increase in the migration of control HUVECs relative to unstimulated cells (Fig. 2d,e). Depletion of CdGAP resulted in a non-significant reduction in basal migration, but led to a 40% decrease in VEGF-stimulated migration when compared to control cells (Fig. 2d,e). Since migration of endothelial cells is essential for angiogenesis, we also evaluated the contribution of CdGAP to the formation of capillaries on Matrigel in response to VEGF (Fig. 2f,g). CdGAP-depleted HUVECs were impaired in their ability to form an interconnected capillary network (Fig. 2f), and a 50% decrease in overall tube length was observed compared to the control condition (Fig. 2g). We further assessed the ability of CdGAP to promote capillary sprouting from collagen-embedded cell spheroids. While VEGF stimulated a robust 3-fold induction of capillary sprouting and extension relative to the unstimulated condition, it was reduced by half in CdGAP-depleted HUVECs (Fig. 2h–j). Overall, these results demonstrate that CdGAP is critical for VEGF-dependent endothelial cell protrusion, migration, and capillary formation.

### CdGAP constitutively interacts with VEGFR2

**Figure 3:**
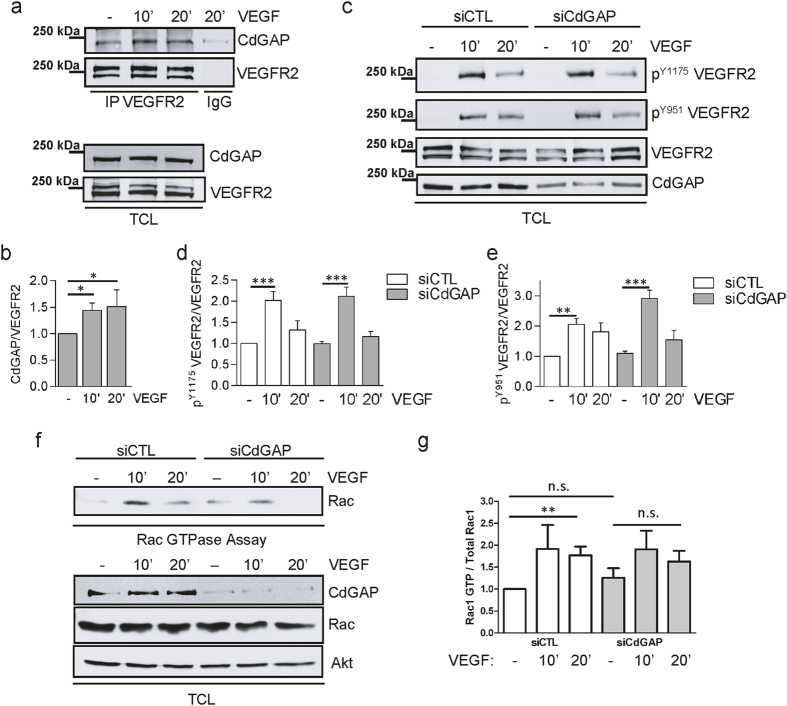
CdGAP associates with VEGFR2 and is required for VEGF-induced Rac1 activation in endothelial cells. **(a)** VEGFR2 immunoprecipitation from lysates of VEGF-stimulated HUVECs. Rabbit IgG was used as a control. Immunoprecipitated (IP) proteins and total cell lysates (TCL) were resolved by SDS-PAGE and immunoblotted for CdGAP and VEGFR2. (**b**) Quantification of CdGAP protein levels co-immunoprecipitating with equal amounts of VEGFR2 by densitometric analysis from (**a**). Error bars indicate SEM (n = 4, *P ≤ 0.05, One-way ANOVA, Newman-Keuls post-test). (**c**) Control and CdGAP-depleted HUVECs were stimulated with VEGF for the indicated times. Total cell lysates (TCL) were resolved by SDS-PAGE and immunoblotted with the indicated antibodies. (**d,e**) Quantification of the ratios of phospho-VEGFR2 on total VEGFR2 protein levels normalized to control, unstimulated cells by densitometric analysis. (n > 3, *P ≤ 0.05, **P ≤ 0.01, ***P ≤ 0.001, n.s., P > 0.05, One-way ANOVA, Bonferroni’s multiple comparisons post-test). (**f**) Control and CdGAP-depleted HUVECs were stimulated with VEGF for the indicated times. GTP-loaded Rac1 was pulled down from protein lysates with GST-CRIB. Rac1-GTP (Rac GTPase assay) and total cell lysates (TCL) were resolved by SDS-PAGE and immunoblotted with the indicated antibodies. Akt was detected as a protein loading control. (**g**) Quantification of the ratios of GTP-bound Rac1 on total Rac1 levels normalized to those of unstimulated control cells (siCTL) by densitometric analysis. Error bars indicate SEM (n = 5, **P ≤ 0.01, n.s., P > 0.05, Unpaired student’s t-test).

Since CdGAP was required for VEGF-induced cell migration and capillary formation, we next examined if CdGAP could associate with and regulate VEGFR2 activation, the main VEGF receptor required for the promotion of these biological processes. HUVECs were stimulated with VEGF for 10 or 20 minutes and VEGFR2 was isolated by immunoprecipitation (Fig. 3a). Subsequent western blotting analysis revealed a basal association of CdGAP with VEGFR2, which was further increased after 10 and 20 minutes of VEGF stimulation (Fig. 3a,b). However, this association did not affect VEGFR2 activation in response to VEGF stimulation, and robust autophosphorylation at Y1175 and Y951 was observed in both control and CdGAP-depleted endothelial cells (Fig. 3c–e). Altogether, these results indicate that CdGAP is part of the VEGFR2 signaling complex in endothelial cells, likely acting downstream of VEGFR2 activation to promote the angiogenic response (Fig. 3c–e).

### VEGF-induced Rac1 activation is impaired in CdGAP-depleted endothelial cells

VEGF-induced endothelial cell motility and capillary formation are in part regulated by the small GTPase Rac1^18^,^38^, and CdGAP has been described as a regulator of Rac1 activity^25^. Thus, we next assessed whether loss of CdGAP affected VEGF-induced Rac1 activation. To achieve this, pull-down assays using the Cdc42/Rac interactive binding (CRIB) domain of PAK fused to GST were performed to assess the levels of active GTP-Rac1 in control and CdGAP-depleted endothelial cells stimulated with VEGF. We observed that VEGF induced a significant 1.8-fold increase in GTP-Rac1 after 20 minutes in control cells (Fig. 3f,g). While CdGAP-depletion in endothelial cells resulted in no significant change in basal levels of active Rac1, VEGF treatment failed to induce a significant Rac1 activation after 10 and 20 minutes (Fig. 3f,g). Therefore, these data reveal that CdGAP is required for the optimal activation of Rac1 during VEGF-mediated signaling in endothelial cells.

### CdGAP depletion impairs VEGF-mediated signaling in endothelial cells

**Figure 4:**
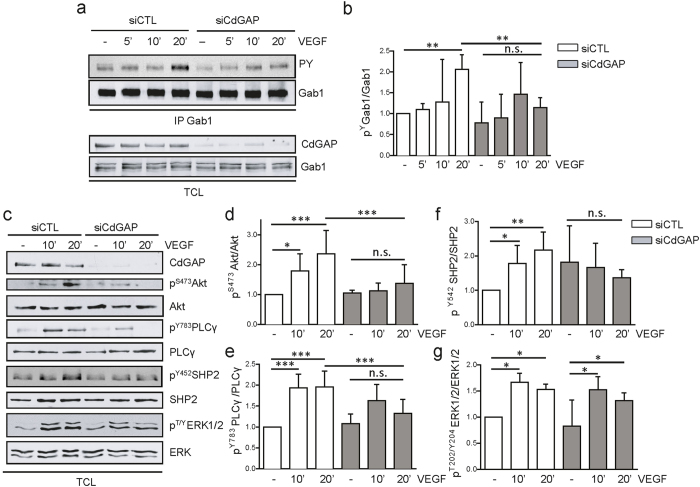
CdGAP is required for VEGF-induced Gab1 tyrosine phosphorylation and downstream signaling in endothelial cells. **(a)** Gab1 was immunoprecipitated (IP) from control and CdGAP-depleted HUVECs stimulated with VEGF for the indicated times. Gab1 IP and total cell lysates (TCL) were resolved by SDS-PAGE and immunoblotted with the indicated antibodies. (**b**) Quantification of the ratios of tyrosine-phosphorylated Gab1 on total Gab1 protein levels normalized to control unstimulated cells by densitometric analysis. Error bars indicate SEM (n = 4, **P ≤ 0.01, n.s., P > 0.05, One-way ANOVA, Newman-Keuls post-test). (**c**) VEGF-dependent cell signaling in control and CdGAP-depleted HUVECs. Total cell lysates (TCL) were resolved by SDS-PAGE and immunoblotted with the indicated antibodies. Quantification of the ratios of phosphoAkt/total Akt (**d**), phosphoPLCγ/total PLCγ (**e**), phosphoSHP2/total SHP2 (**f**), and phosphoERK1/2/total ERK1/2 (**g**) normalized to control unstimulated cells by densitometric analysis. Error bars indicate SEM (n = 5, *P ≤ 0.05, **P ≤ 0.01, ***P ≤ 0.001, n.s., P > 0.05, One-way ANOVA, Newman-Keuls post-test).

We next explored the contribution of CdGAP to VEGF-induced signaling pathways in endothelial cells. VEGFR2-dependent tyrosine phosphorylation of the scaffolding adapter protein Gab1 mediates its association with signaling proteins, including PI3K, SHP2, and PLCγ^12^,^13^, and this contributes to their optimal activation/phosphorylation, as well as to the downstream activation of the pro-angiogenic mediators Akt and ERK1/2^12^,^13^,^14^,^15^,^16^. To determine if CdGAP was involved in the regulation of these VEGF-induced signaling pathways, control and CdGAP-depleted HUVECs were stimulated with VEGF for 5 to 20 minutes, and the tyrosine phosphorylation level of immunoprecipitated Gab1 was assessed by western blotting. In control HUVECs, tyrosine phosphorylation of Gab1 was maximally increased after 20 minutes of VEGF stimulation, while it was significantly reduced in CdGAP-depleted cells (Fig. 4a,b). Consistent with this, the VEGF-dependent phospho-activation of Akt was strongly inhibited in CdGAP-depleted cells (Fig. 4c,d), and the phosphorylation of SHP2 and PLCγ was also significantly decreased(Fig. 4c,e,f). In contrast, the downstream activation of ERK1/2 was not affected in these conditions (Fig. 4c,g). Taken together, these results demonstrate that CdGAP is essential for VEGF-mediated phosphorylation of Gab1, a central intracellular signaling intermediate, and for the optimal downtream activation and/or phosphorylation of Akt and SHP2, and to a lesser extent of PLCγ, during endothelial cell migration and angiogenesis.

## Discussion

The *CdGAP* gene was reported as one of the 17 RhoGAPs highly expressed in HUVECs and in the developing vascular system of zebrafish^24^,^27^, but until now, its contribution to vascular endothelial cell biology remained unexplored. In this study we demonstrate for the first time that CdGAP is critical for VEGF-induced angiogenic signaling in human endothelial cells, and correlate this with a function for CdGAP in mammalian vascular development *in vivo*. CdGAP-deficient embryos are hypovascularized and present vascular defects such as hemorrhages, which lead to edema and may be in part responsible for the partial embryonic/perinatal lethality observed in CdGAP-deficient mice. CdGAP is crucial for sprouting angiogenesis from aortic explants and from endothelial cell spheroids, for *in vitro* capillary formation, and for endothelial cell migration in response to VEGF. Surprisingly, depletion of CdGAP does not lead to an increase in Rac1 activation, and this may be explained by impaired VEGF signaling in CdGAP-depleted cells. Overall, our work describes an essential regulation of angiogenesis by CdGAP, as evidenced by impaired sprouting and attenuated regulation of endothelial cell motility and capillary formation in response to VEGF.

We show that CdGAP associates with VEGFR2 and promotes VEGF-triggered activation of downstream signaling pathways essential for optimal Rac1 activation and angiogenesis. Remarkably, CdGAP tightly controls the tyrosine phosphorylation of Gab1, an important mediator of actin reorganization and an enhancer of the PI3K-Akt and SHP2-dependent signaling pathways essential for endothelial cell migration and capillary formation in response to VEGF^12^,^13^,^16^,^39^. Similarly to CdGAP, Gab1 is highly expressed in blood vessels and limbs at mid-gestation, and Gab1-deficient mice also display vascular defects and hemorrhages^40^. Moreover, Gab1 and Akt1 are critical for VEGF- and ischemia-induced angiogenesis *in vivo*^15^,^16^,^17^,^41^,^42^, emphasizing the molecular link between CdGAP, VEGF intracellular mediators, and vascular functions. The observed genetic association between the *CdGAP* gene and an increased risk for coronary artery disease also supports a role for CdGAP in endothelial cell biology^43^. Collectively, these data strongly suggest that the altered function of CdGAP may potentially contribute to the development of a wide range of vascular and cardiac dysfunctions.

Recently, mutations in the *CdGAP* gene have been identified in families of patients with autosomal dominant AOS^33^,^34^,^44^,^45^. AOS is a heterogeneous disorder, typically characterized by the presence of both aplasia cutis congenita of the scalp vertex and terminal limb defects^35^,^36^. Additionally, vascular and cardiac anomalies, including cutis marmorata telangiectatica and pulmonary hypertension, have been observed in AOS-affected patients^35^,^46^. Although no cardiovascular malformations were observed in the identified AOS patients carrying heterozygous gain-of-function mutations in the CdGAP gene^33^,^34^,^46^, our results demonstrate here that the complete loss of CdGAP expression in mouse embryos leads to hypovascularization, vascular defects and incomplete embryonic/perinatal lethality. Interestingly, mutations in the *DOCK6* gene encoding a Rac1/Cdc42 guanine nucleotide exchange factor responsible for an autosomal-recessive variant of AOS were recently associated with impaired vascular functions, supporting the importance of Rac1/Cdc42 signaling processes in vascular development^45^,^47^. Furthermore, mutations in several genes of the Notch signaling pathway, including EOGT, RBPJ, Notch, and the Notch ligand Dll4, that encode important regulators of vascular development^48^,^49^,^50^ were recently identified in AOS patients^51^,^52^,^53^,^54^. Together with our findings, these recent genetic studies converge towards two major altered signaling pathways in the etiology of the disorder, the Notch and Cdc42/Rac1 pathways, which may be interconnected to control the development of the embryonic vasculature^55^. Notably, it was reported that VEGF modulates Dll4 expression via the ERK/Akt-dependent inactivation of GSK3β^50^, and CdGAP, which is also phosphorylated and inactivated by GSK3β^56^, interacts with VEGFR2 to regulate VEGF-induced Akt activation. Consequently, CdGAP might indirectly support the Dll4/Notch axis to control vascular development, providing a molecular link between the altered Notch and CdGAP pathways in the pathogenesis of AOS. In summary, these findings illustrate a critical role for CdGAP in angiogenesis and VEGF signaling, and provide novel mechanistic insights into the molecular causes of AOS.

## Methods

### Antibodies and Reagents

Antibodies against phospho-tyrosine (PY99), Gab1 (Clone H-198), and VEGFR2 (C-1158) were purchased from Santa Cruz Biotechnology. Anti-p^S473^ Akt, Akt, p^Y783^ PLCγ, PLCγ, p^T202/Y204^ERK1/2, ERK1/2, p^Y1175^ VEGFR2, p^Y951^ VEGFR2, Horseradish peroxidase (HRP)-conjugated anti-mouse or anti-rabbit IgGs were purchased from Cell Signalling. Antibodies against Rac1 and Matrigel were purchased from BD Biosciences. CdGAP antibody (HPA036380) was purchased from Sigma. VEGFR2 mouse antibody used for western blotting was purchased from Upstate (Clone CH-11). Gelatin type B was from Fisher Scientific. Recombinant human VEGF-A was obtained from the Biological Resources Branch Preclinical Repository of the National Cancer Institute - Frederick Cancer Research and Development Center. Transwell filters (poly-carbonate membrane, 8 μm pore-size) were from Corning.

### Generation of CdGAP^fl/fl^ and CdGAP-deficient mice

The F1 CdGAP^fl/+^ mice were generated by InGenious Targeting Laboratory, inc. For targeting CdGAP, a single loxP site containing an engineered EcoRV sequence was inserted 177bp 5′ of exon 1, and a loxP/FRT flanked Neo cassette containing an engineered Spe1sequence was inserted 270bp 3′ of exon 1. F1 CdGAP^fl/+^ mice were in a mixed (*C57BL/6 *× *129/SvEv*) hybrid genetic background. F1 CdGAP^fl/+^ mice were crossed with mice expressing FLP recombinase (Jackson Laboratories) to delete the Neo cassette from the germline. CdGAP^−/−^ mice were generated by crossing F4 CdGAP^fl/fl^ mice with mice expressing Cre recombinase under the Meox2 promoter (a gift from N. Saidah, Institut de Recherches Cliniques de Montreal), which is active as early as embryonic day 5 in epiblast-derived tissues^37^. CdGAP^fl/−^; Meox2-Cre^−^ were intercrossed to generate CdGAP^−/−^; Meox2-Cre^−^ used in the study. CdGAP^fl/fl^; Meox2-Cre^−^ were used as controls (Supplementary Fig. 2). All experiments were performed using age, sex and littermate genetic controls. Genotyping was performed using DNA isolated from mouse tails collected upon weaning or from mouse embryos at E15.5 using the following locus-specific primer sets to detect the exon 1 deleted allele: 5′CCTGCGCTGTGCAAAGAGCCT3′; 5′CCCAAGTTTAAGACCCGAGCTCC3′; and to detect the floxed allele: 5′GAGCAATTCCATGAGCACCCATC3′; 5′AGGTTGGAAATTTTTGGCAGCTGT3′. The presence of the Cre allele under the meox2 promoter was assessed by using the primers and protocol provided by Jackson Laboratories. All animals were cared for in accordance with guidelines established by the McGill University Animal Care and Use Committee. All experimental procedures (protocol no. 2009-5648) were approved by the McGill University Animal Care and Use Committee.

### Histology

E15.5 embryos were dissected and briefly visualized under a dissecting microscope (Zeiss Stemi 2000-C) while pictures were taken using a Zeiss Axiocam MRc camera. Embryos were then fixed in 4% paraformaldehyde in PBS overnight at 4 °C. The next day, embryos were washed 3 times with PBS and either cryoprotected in 30% sucrose at 4 °C for 3 days or paraffin-embedded and sectioned. Cryoprotected embryos were then embedded in optimum cutting temperature compound and snap frozen in 2-methyl butane at −30 °C and stored at −80 °C until sectioned. Sections were then processed following standard H&E protocols. Paraffin-embedded embryos were sectioned at 5 μm/section while cryosections were 14 μm-thick. Sections were visualized using a Zeiss Imager M2 upright microscope and images obtained with a Zeiss AxioCam ICc5 camera.

### Mouse aortic ring assay

Mouse aortic rings were prepared as previously described^57^. Briefly, thoracic aortas of 6-week old CdGAP^fl/fl^ and CdGAP^−/−^ mice were dissected and 0.5 mm rings were serum-starved overnight in OptiMEM (Gibco Life Technologies). Rings were then embedded in Matrigel and cultured in OptiMEM 2.5% FBS with PBS as a control or VEGF (50 ng/ml). Medium was changed on day 4. At day 7, rings were photographed using the Axio Observer.Z1 microscope (Zeiss) and the AxioCam HRm camera (Zeiss) with a 10x objective lens. Images were captured with Axiovison 4.8.2 software (Zeiss). The area covered by sprouts was evaluated using the Metamorph software (Molecular Devices).

### Cell Culture and nucleofection

Human umbilical vein endothelial cells (HUVECs; from Cascade Biologics/Invitrogen) were cultured (passages 1 to 4) on 0.2% gelatin-coated tissue culture dishes (Corning), and maintained in M200 medium (Invitrogen) supplemented with low serum growth supplement LSGS (Gibco; Cascades Biologics; Invitrogen), 2% fetal bovine serum, 1 μg/ml hydrocortisone, 10 ng/ml human epidermal growth factor, 3 ng/ml basic fibroblast growth factor, 10 μg/ml heparin, and 50 μg/ml gentamycin (Wisent). Downregulation of endogenous CdGAP expression was achieved by electroporating between 5 × 10^5^ and 5 × 10^6^ HUVECs at passage 4 with 20 nM of either non-targeting No2 siRNAs (Dharmacon) or CdGAP siRNAs from Ambion (GGAGUCACCUCAAACAUACtt sense, GUAUGUUUGAGGUGACUCCtg anti-sense)^29^ using the Nucleofector II (program U- 001; Lonza) and the Amaxa HUVEC Nucleofector Kit following the manufacturer’s recommendations. Forty-eight hours after nucleofection, cells were either serum-starved before VEGF stimulation and lysed, or processed for the various *in vitro* biological assays.

### Migration Assay

Nucleofected HUVECs (1 × 10^5^ cells in 200 μl of serum-free M200 medium) were seeded on Transwell filters previously coated for 2 h with 0.2% Gelatin, and inserted in 24-well plates containing 500 μl of serum-free M200 medium with or without VEGF(10 ng/ml). At the end of the assay (6 h), cells were fixed with phosphate-buffered formalin for 20 min and stained with crystal violet (0.1% in 20% methanol). Cells remaining on the upper surface of the filter were wiped off and those that migrated through the filter were visualized and counted (a minimum of 6 fields/insert at 10x magnification) with a Nikon eclipse TE300 microscope and photographed with a Nikon digital sight DS FI2 camera.

### Spheroid assay

This assay was based on previously described protocols^58^. Briefly, cells (1 × 10^3^) nucleofected with control or CdGAP siRNAs were immediately cultured in supplemented M200 medium and 0.5% methyl cellulose as hanging drops (15 μl) for 36 h. Spheroids were next collected by centrifugation (200 g, 3 min) and 50–75 spheroids were resuspended in 500 μl of complete medium containing 0.5% of methyl cellulose. The suspension was added to an equal volume of a collagen solution (250 μl of home-made rat tail collagen^59^, 100 μl NaOH 0.1 M, 50 μl PBS 10X, 100 μl Dulbecco’s modified Eagle medium (DMEM) and 50 ng/ml of VEGF or PBS as a control) and mixed thoroughly on ice. One ml of this mix was deposited per well in a pre-heated 24-well plate and collagen was allowed to solidify at 37 °C for 10 minutes. DMEM (30 μl) was added to each well to prevent drying. Spheroids were incubated for 6–8 h and then imaged using the Zeiss Axio Observer.Z1 microscope. Photographs were taken with the AxioCam HRm (Zeiss) camera using a 20x objective lens. Images were captured with the Axiovison 4.8.2 software (Zeiss). The number and total length of all sprouts were determined for at least 10 spheroids per condition using the Metamorph software (Molecular Devices).

### Capillary formation assay

This assay was performed as described in^60^. Briefly, 48 h after nucleofection of siRNAs, HUVECs (2 × 10^4^ cells/wells) were seeded in duplicates on solidified Matrigel (BD Biosciences) in flat-bottom 96-well plates (50 μl/well) and incubated for 4–6 h at 37 °C in non-supplemented M200 medium containing VEGF (50 ng/ml). Pictures were taken using a Nikon eclipse TE300 microscope and photographed with a Nikon digital sight DS FI2 camera using a 20x objective lens. The length of assembled capillaries was quantified using the Metamorph Software.

### Cell stimulation, lysis, immunoprecipitation, immunoblotting and Rho GTPase Assays

Following starvation in M200 medium for 6 h, HUVECs were stimulated with VEGF (50 ng/ml) at 37 °C for the indicated times and incubated in PBS containing 1 mM Na_3_VO_4_ for 30 min on ice. Cells were next lysed in a 50 mM HEPES (pH 7.5) lysis buffer containing 0.5% Nonidet P-40, 0.5% Triton X-100, 10% glycerol, 150 mM NaCl, 1 mM EDTA, 1 mM PMSF, 10 μg/ml aprotinin, 10 μg/ml leupeptin, 5 mM NaF and 1 mM Na_3_VO_4_ for 20 minutes. The quantification of Rac1 activity was performed as previously described^61^. Briefly, equal amounts of protein lysates (120 μg) were incubated for 1 h at 4 °C with 15 μg of the PAK CRIB domain fused to GST (GST-CRIB) and coupled to glutathione-Sepharose beads. Beads were washed 3 times with binding buffer containing 1% NP-40, followed by 2 washes with binding buffer without NP-40. Gab1 and VEGFR2 were immunoprecipitated from HUVEC protein lysates (300 μg and 1 mg, respectively) O/N at 4 °C with 2 μg of anti-Gab1 and 4 μg of anti-FLK-1 rabbit antibodies, and further incubated for 3h with 30 μl of a 50% suspension of Protein A-coupled Sepharose beads (Amersham Biosciences/GE Healthcare). Immunoprecipitated/pull-downed proteins or total protein extracts (50 μg) were subjected to SDS-PAGE and transferred onto nitrocellulose membranes (0.45 μm) (Bio-Rad). Western blotting was carried out using appropriate HRP-conjugated secondary antibodies and chemiluminescence-based detection systems according to the manufacturer’s recommendations (ECL detection reagent from Amersham, or Visualizer kit from Millipore).

### Data Analysis

Statistical analysis was performed with GraphPad Prism 6 software. P-values of less than 0.05 were considered to be significant (*P ≤ 0.05 **P ≤ 0.01 ***P ≤ 0.001). Densitometric analyses were performed with the Quantity One 4.6.3 software (Bio-Rad) and are respresented as the mean of at least 3 independent experiments. Error bars indicate the SEM.

## Additional Information

**How to cite this article**: Caron, C. *et al*. CdGAP/ARHGAP31, a Cdc42/Rac1 GTPase regulator, is critical for vascular development and VEGF-mediated angiogenesis. *Sci. Rep.* **6**, 27485; doi: 10.1038/srep27485 (2016).

## Supplementary Material

Supplementary Information
