# Supplementary material for: CdGAP/ARHGAP31, a Cdc42/Rac1 GTPase regulator, is critical for vascular development and VEGF-mediated angiogenesis
Source: Sci Rep. 2016 Jun 7;6:27485. doi: 10.1038/srep27485 (PMC4895392; doi:10.1038/srep27485)
Supplement: Supplementary Information [file srep27485-s1.pdf]

Supplementary Figures

CdGAP/ARHGAP31, a Cdc42/Rac1 GTPase regulator, is critical for vascular development and VEGF-mediated angiogenesis

Christine Caron, Jonathan DeGeer, Patrick Fournier, Philippe M. Duquette, Vilayphone Luangrath, Hidetaka Ishii, Fereshteh Karimzadeh, Nathalie Lamarche-Vane and Isabelle Royal

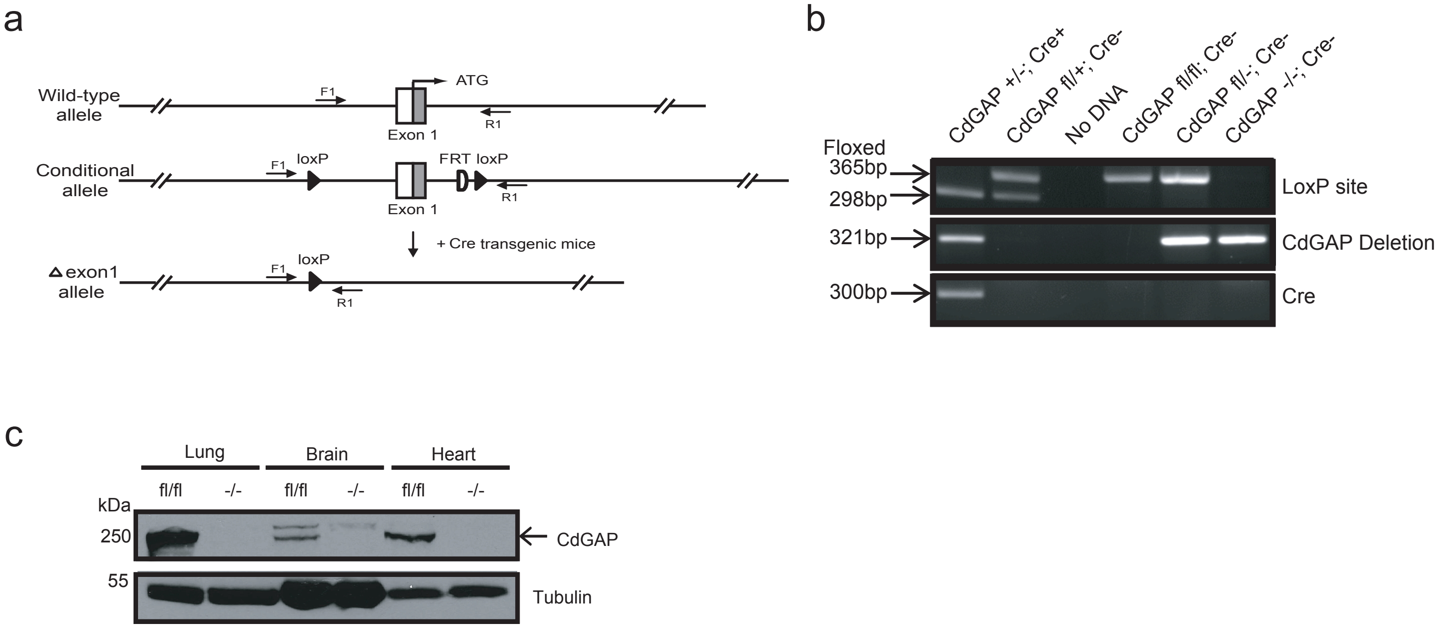

Supplementary Figure 1. Generation of CdGAP$^{-/-}$ mice. (**a**) Schematic drawing of the targeting strategy for the production of *CdGAP* conditional floxed (fl) mice. Primers (F1 and R1) for PCR assessment of *CdGAP* exon1 deletion (Δexon1). (**b**) CdGAP$^{-/-}$ mice were generated by crossing CdGAP$^{fl/fl}$ mice with mice expressing Cre recombinase under the Meox2 promoter. Samples for PCR were prepared from mouse tails. Wild-type (+) allele, 298 bp; conditional allele-specific (fl), 365 bp; CdGAP deletion (-), 321 bp; Cre allele, 300 bp. (**c**) Western blot analysis of CdGAP expression was performed on lung, brain, and heart tissue lysates from CdGAP$^{fl/fl}$ and CdGAP$^{-/-}$ mice. Tubulin was used as a protein loading control. CdGAP was absent in all CdGAP$^{-/-}$ tissue tested.

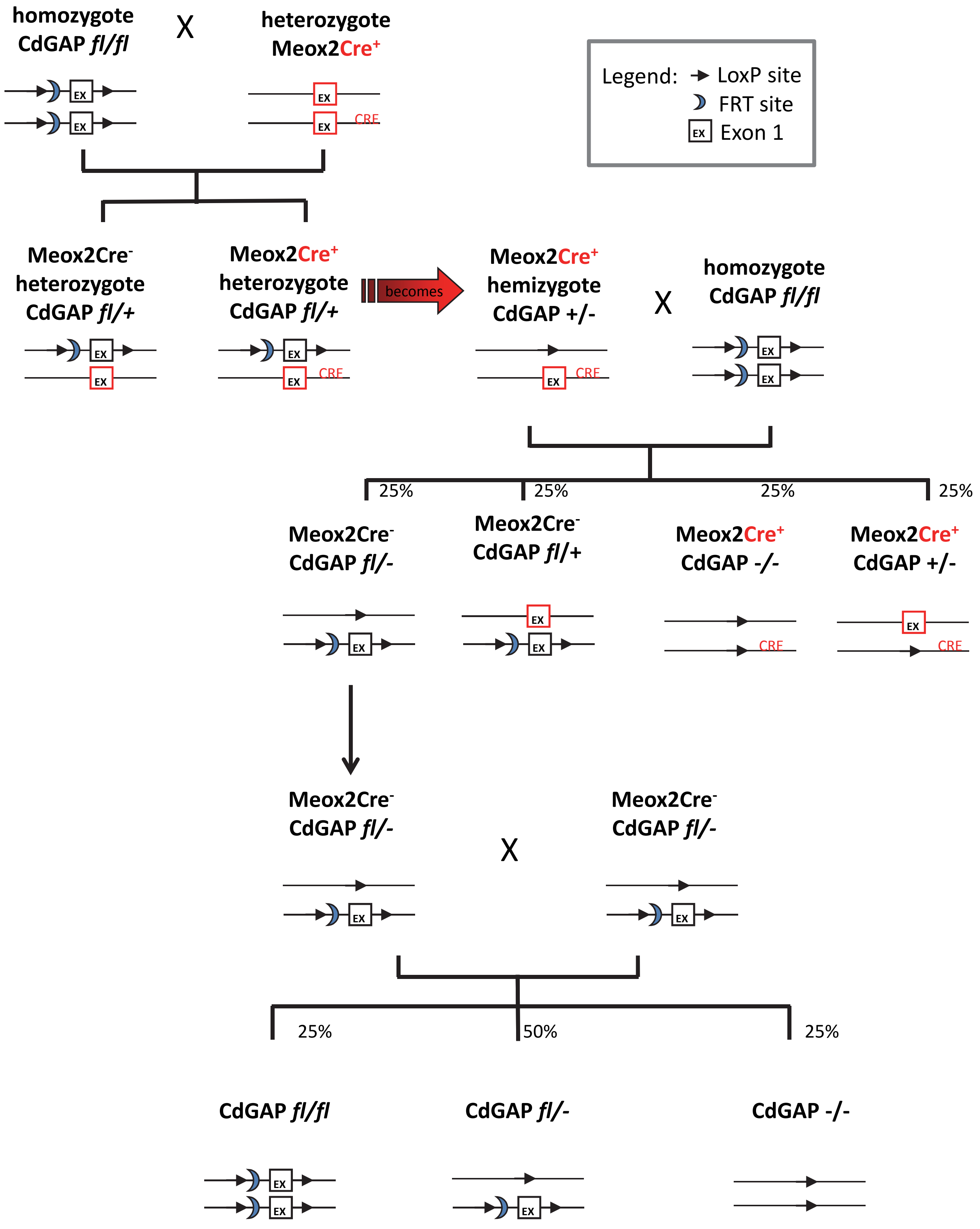

Supplementary Figure 2. Breeding scheme of the $CdGAP^{fl/fl}$ mice crossed with mice expressing Cre recombinase under the Meox2 promoter to generate $CdGAP^{-/-}$ mice.
